# Intracranial Calcification Masquerading as Hemorrhage in a Patient with Multiple Myeloma Presenting with Facial Neuropathy

**DOI:** 10.7759/cureus.2841

**Published:** 2018-06-19

**Authors:** Naresh Mullaguri, Anusha Battineni, Raviteja Guddeti

**Affiliations:** 1 Cerebrovascular Center, Cleveland Clinic Foundation, Cleveland, USA; 2 Neurology, Cleveland Clinic Foundation, Cleveland, USA; 3 Cardiovascular Medicine, Creighton University School of Medicine, Omaha, USA

**Keywords:** multiple myeloma, calcification, intracerebral hemorrhage, facial nerve palsy

## Abstract

Multiple myeloma is an immunoglobulin-producing plasma cell neoplasm that commonly affects the bones, kidneys, the hematopoietic system, and rarely the nervous system. Peripheral nervous system involvement in the form of cranial neuropathy, radiculopathy, and polyneuropathy are common. Compressive myelopathy constitutes the majority of central nervous system disorders followed by cerebrovascular disorders, intracranial plasmacytomas, and leptomeningeal myelomatosis. Cerebrovascular complications such as acute ischemic stroke and transient ischemic attack are not uncommon. Intracerebral hemorrhage, although infrequent, can be secondary to refractory hypertension from renal failure and intratumoral hemorrhage in intracerebral plasmacytomas. Metastatic calcifications in lungs, liver, and skin with high levels of serum calcium and phosphate are seen in patients with multiple myeloma, but intracerebral calcifications are not common. We report an unusual case of intracranial calcification masquerading as acute intracerebral hemorrhage in a patient presenting with acute facial weakness.

## Introduction

Multiple myeloma (MM) is an infiltrative neoplastic disease of immunoglobulin-producing plasma cells. It commonly affects the bones, kidneys, and hematopoietic system. Bone osteolytic lesions can cause compression fractures and hypercalcemia [1-2]. The peripheral nervous system is commonly involved in MM. Thoracic and lumbosacral radiculopathy develop due to paravertebral plasmacytoma and vertebral collapse. Peripheral neuropathy is usually rare secondary to immunoglobulin light chain amyloidosis except in polyneuropathy, organomegaly, endocrinopathy, monoclonal protein, and skin changes (POEMS) syndrome where its prevalence is 100 percent [3]. MM can affect the central nervous system with myelopathy being the most common manifestation at an estimated prevalence of five percent and is considered a medical emergency. The most common causes include extramedullary plasmacytoma or compression fracture of the vertebra due to osteolytic lesions [3]. Intracranial plasmacytomas and leptomeningeal myelomatosis are rare and associated with poor prognosis. Rare cases of encephalopathy secondary to hyperviscosity syndrome and elevated ammonia levels have been reported in the literature. Intracerebral calcifications are rare despite the increased prevalence of hypercalcemia in MM, and it can mimic acute intracerebral hemorrhage if present in the area of clinical interest. We report a patient with acute onset facial weakness with intracranial calcification on computerized tomography (CT) masquerading as acute intracerebral hemorrhage.

## Case presentation

A 75-year-old Caucasian male presented to the emergency room with the chief complaint of left-sided facial weakness. He noticed the weakness while brushing his teeth, and also noted slurred speech. He denied eye pain, tearing or redness, hearing loss, difficulty with swallowing, and weakness or numbness in his extremities. He was able to understand and express his words without any difficulty. He denied any headache, gait problems, falls or recent illness, and prior history of stroke or seizures. Past medical history was positive for hypertension, hyperlipidemia, diabetes mellitus, and multiple myeloma (MM). He was on chemotherapy with ixazomib, acyclovir prophylaxis, and a daily aspirin. Vital signs were unremarkable. Physical examination was significant for lower facial muscle weakness with no difficulty in closing the left eye. His National Institutes of Health (NIH) stroke scale was two for facial weakness and dysarthria.

**Figure 1:**
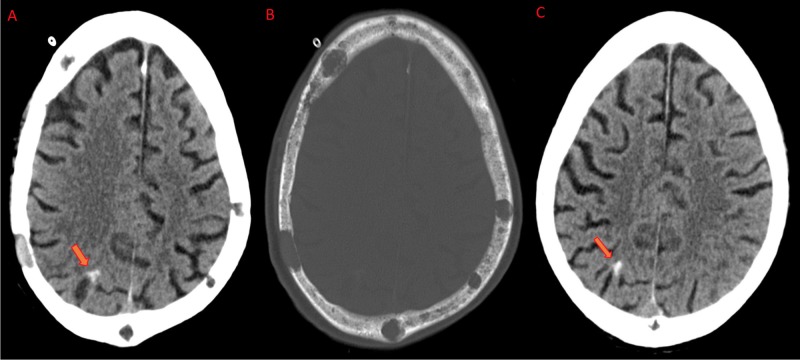
Computerized tomography (CT) axial images A) On the day of admission showing right parietal cortex hyperdense (arrow) lesion with no perilesional edema; B) Skull showing well demarcated, punched out, lytic lesions of multiple myeloma; C) Follow-up CT of brain showed persistent calcification.​

His blood work was remarkable for mild anemia and normal serum calcium. Coagulation workup was unremarkable. A non-contrast computerized tomography (CT) of the head showed acute intraparenchymal hemorrhage in the right parietal region with global cerebral atrophy (Figure 1A-1B), (repeat CT of the brain four weeks later showed the same hyperdensity; see Figure 1C).

**Figure 2:**
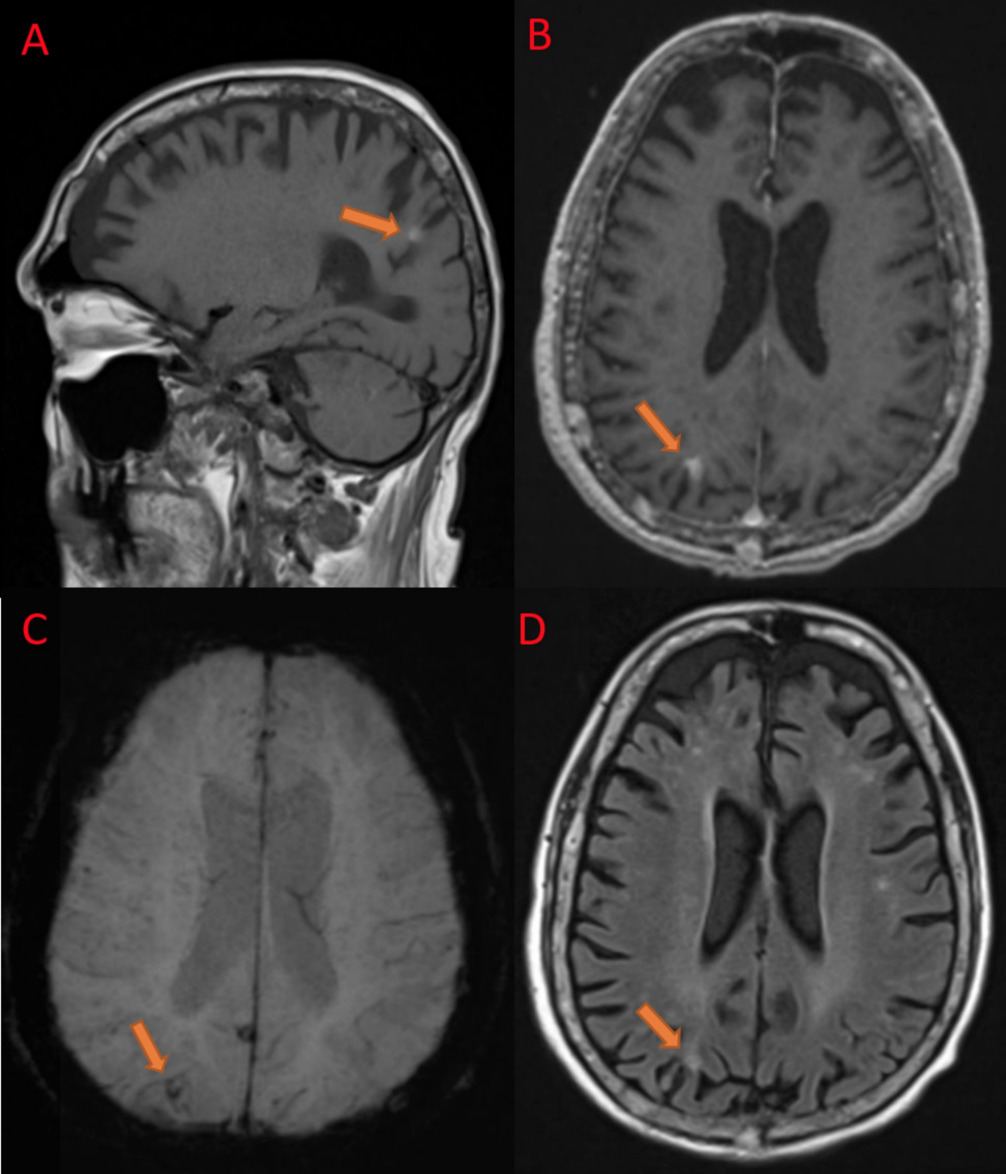
Magnetic resonance imaging of the Brain A) Sagittal T1 weighted image showing hyperintensity in the right parietal region (arrow), B) Axial T1 weighted post-contrast image showing no significant enhancement compared to T1-weighted image (arrow), C) Axial susceptibility-weighted Image (SWI) showing small blooming artifact in the same location (arrow), and D) Axial T2-weighted fluid attenuation and inversion recovery (FLAIR) scan showing hyperintensity (arrow) (seen in late subacute hemorrhage).

However, CT angiogram of the head and neck was unremarkable. On day two, he was unable to close his left eye with Bell’s phenomenon. The differential diagnosis considered were lower motor neuron (LMN) facial palsy from multiple myeloma or diabetes mellitus or focal seizure from acute right parietal intracerebral hemorrhage. Magnetic resonance imaging (MRI) of the brain showed hyperintensity in T1-weighted sequence (Figure 2A), no significant post-contrast enhancement (Figure 2B), blooming artifact in the susceptibility weighted imaging (SWI) (Figure 2C), and hyperintensity in T2-weighted fluid attenuation and inversion recovery (FLAIR) test (Figure 2D) consistent with possible right parietal hemorrhage or calcification.

Varicella zoster antibody test was negative. Monoclonal protein analysis showed an atypical restricted band in the lambda region consistent with monoclonal gammopathy. Given the LMN type of facial weakness, we provisionally diagnosed him with Bell’s palsy. Focal seizure was clinically ruled out as a possibility due to persistent facial weakness with no fluctuation. We started him on valacyclovir 1000 mg three times daily for seven days with a subsequent switch to his home oral acyclovir chemoprophylaxis for multiple myeloma (400 mg by mouth twice daily) and oral prednisone taper. At the four weeks follow-up appointment, his symptoms improved to a mild residual weakness of the left lower face, but he was able to close his eyes completely. Follow-up CT of the brain showed the same hyperdensity in the right parietal region consistent with intracerebral calcification without surrounding edema (Figure 1C).

## Discussion

Multiple myeloma (MM) is a plasma cell infiltrative neoplastic disorder producing monoclonal immunoglobulin. MM predominantly affects bones, kidneys, and hematopoietic tissue, but it can also involve the nervous system [1-2]. A broad spectrum of neurological diseases can occur in MM due to mechanical compression of neural structures by plasmacytomas, infiltration, secondary amyloidosis from light chains, metabolic abnormalities like hyperviscosity, hypercalcemia, uremia, and during chemotherapy [3]. Compressive radiculopathy and myelopathy constitute about 10% of MM patients with neurological involvement. Cranial nerve involvement in MM is secondary to skull base plasmacytomas, leptomeningeal extension, and intraparenchymal involvement. Most commonly, the trigeminal, sixth, and eighth cranial nerves are affected due to plasmacytomas affecting the mandible (numb-chin syndrome), petrous bone, and the sella respectively [3-4].

Cerebrovascular complications in MM are rare. In a single-center case-control study by Hinduja et al., about four percent of MM patients had a stroke, of which acute ischemic stroke or transient ischemic attack (TIA) constituted 96 percent, and intracerebral hemorrhage constituted four percent [5]. Although rare, intracerebral plasmacytomas can arise from meninges and cerebral parenchyma and share radiological features with intracerebral hemorrhage with hyperdensity on CT imaging [6-9]. However, they are also characterized by intense contrast enhancement, peritumoral edema, and rarely intratumoral hemorrhage [10-13]. Isolated light chain deposition in the cerebral blood vessels can present with recurrent hemorrhage simulating cerebral amyloid angiopathy without evidence of systemic disease [14]. MRI can be helpful to differentiate between calcification and acute hemorrhage depending upon its appearance in diffusion-weighted imaging (DWI) and T1 and T2-weighted sequences. In our patient, there was no post-contrast enhancement or perilesional edema on MRI, and this ruled out the possibility of intracerebral plasmacytoma. Hyperintensity in both T1 and T2-weighted sequences suggest late subacute hemorrhage which did not fit with the acute clinical presentation. 

Metastatic calcification is a well-known paraneoplastic complication of MM. Lungs, liver, and skin are frequently affected due to increased levels of serum calcium and phosphate [15-16]. Calcification of basal ganglia (Fahr's disease) and cerebellar nuclei have been reported in the literature, but not cortical calcifications [17-18]. In an acute setting, it is difficult to differentiate between calcification and hemorrhage in neuroimaging (CT scan of the brain), and this poses a challenge to the physician if the lesion is in the area of clinical interest as in our patient [19]. Misdiagnosis of calcification as hemorrhage can lead to hospitalization and unnecessary investigations which can be avoided with careful clinical localization and neuroimaging features of the lesion in most cases. Given the characteristics of lower motor neuron facial palsy, the lesion localized to parietal cortex, and neuroimaging, we presumptively diagnosed it as an intracranial calcification and confirmed the finding in the follow-up imaging.

## Conclusions

Intracerebral calcifications can sometimes masquerade as parenchymal hemorrhage leading to a misdiagnosis. Careful clinical localization and neuroimaging characteristics can help differentiate these two entities. Multiple myeloma with intracranial calcification is rare, and caution must be exercised when interpreting neuroimaging studies.
